# RoB-SPEO: A tool for assessing risk of bias in studies estimating the prevalence of exposure to occupational risk factors from the WHO/ILO Joint Estimates of the Work-related Burden of Disease and Injury

**DOI:** 10.1016/j.envint.2019.105039

**Published:** 2020-02

**Authors:** Frank Pega, Susan L. Norris, Claudine Backes, Lisa A. Bero, Alexis Descatha, Diana Gagliardi, Lode Godderis, Tom Loney, Alberto Modenese, Rebecca L. Morgan, Daniela Pachito, Marilia B.S. Paulo, Paul T.J. Scheepers, Vivi Schlünssen, Daria Sgargi, Ellen K. Silbergeld, Kathrine Sørensen, Patrice Sutton, Thomas Tenkate, Denise Torreão Corrêa da Silva, Yuka Ujita, Emilie van Deventer, Tracey J. Woodruff, Daniele Mandrioli

**Affiliations:** a Department of Public Health, Environmental and Social Determinants of Health, World Health Organization, Geneva, Switzerland; b Department of Information, Evidence and Research, World Health Organization, Geneva, Switzerland; c Charles Perkins Centre, The University of Sydney, Sydney, Australia; d AP-HP (Paris Hospital “Assistance Publique Hôpitaux de Paris”), Occupational Health Unit, University Hospital of West Suburb of Paris, Poincaré Site, Garches, France; e Versailles St-Quentin Univ - Paris Saclay Univ (UVSQ), UMS 011, UMR-S 1168, France; f Inserm, U1168 (VIMA: Aging and chronic diseases. Epidemiological and public health approaches), UMS 011 (Population-based Epidemiologic Cohorts Unit), Villejuif, France; g Inail, Department of Occupational and Environmental Medicine, Epidemiology and Hygiene, Rome, Italy; h Centre for Environment and Health, KU Leuven, Leuven, Belgium; i KIR Department (Knowledge, Information & Research), IDEWE, External Service for Prevention and Protection at Work, Leuven, Belgium; j Institute of Public Health, College of Medicine and Health Sciences, United Arab Emirates University, Al Ain, United Arab Emirates; k College of Medicine, Mohammed Bin Rashid University of Medicine and Health Sciences, Dubai, United Arab Emirates; l Department of Biomedical, Metabolic and Neural Sciences, University of Modena and Reggio Emilia, Modena, Italy; m Department of Health Research Methods, Evidence and Impact, McMaster University, Ontario, Canada; n Evidence-based Health, Universidade Federal de São Paulo, Sao Paulo, Brazil; o Cochrane Brazil, Sao Paulo, Brazil; p Institute of Public Health, College of Medicine & Health Sciences, United Arab Emirates University, Al Ain, United Arab Emirates; q Global Health and Tropical Medicine, Instituto de Higiene e Medicina Tropical, Universidade Nova de Lisboa, Lisbon, Portugal; r Radboud Institute for Health Sciences, Radboudumc, Nijmegen, the Netherlands; s Department of Public Health, Aarhus University, Aarhus, Denmark; t National Research Center for the Working Environment, Copenhagen, Denmark; u Cesare Maltoni Cancer Research Center, Ramazzini Institute, Bologna, Italy; v Department of Environmental Health and Engineering, Johns Hopkins University Bloomberg School of Public Health, Baltimore, MD, United States of America; w National Research Centre for the Working Environment, Copenhagen, Denmark; x Program on Reproductive Health and the Environment, University of California San Francisco, San Francisco, United States of America; y School of Occupational and Public Health, Ryerson University, Toronto, Ontario, Canada; z Workers' Health and Human Ecology Research Center, National School of Public Health Sergio Arouca, Oswaldo Cruz Foundation, Rio de Janeiro, RJ, Brazil; aa Labour Administration, Labour Inspection and Occupational Safety and Health Branch, International Labour Organization, Geneva, Switzerland

## Abstract

**Background:**

The World Health Organization (WHO) and the International Labour Organization (ILO) are developing joint estimates of the work-related burden of disease and injury (WHO/ILO Joint Estimates). For this, systematic reviews of studies estimating the prevalence of exposure to selected occupational risk factors will be conducted to provide input data for estimations of the number of exposed workers. A critical part of systematic review methods is to assess risk of bias (RoB) of individual studies. In this article, we present and describe the development of such a tool, called the Risk of Bias in Studies estimating Prevalence of Exposure to Occupational risk factors (RoB-SPEO) tool; report results from RoB-SPEO's pilot testing; note RoB-SPEO's limitations; and suggest how the tool might be tested and developed further.

**Methods:**

Selected existing RoB tools used in environmental and occupational health systematic reviews were reviewed and analysed. From existing tools, we identified domains for the new tool and, if necessary, added new domains. For each domain, we then identified and integrated components from the existing tools (i.e. instructions, domains, guiding questions, considerations, ratings and rating criteria), and, if necessary, we developed new components. Finally, we elicited feedback from other systematic review methodologists and exposure scientists and agreed upon RoB-SPEO. Nine experts pilot tested RoB-SPEO, and we calculated a raw measure of inter-rater agreement (*P*_*i*_) for each of its domain, rating *P*_*i*_ < 0.4 as poor, 0.4 ≤ *P*_*i*_ ≥ 0.8 as substantial and *P*_*i*_ > 0.80 as almost perfect agreement.

**Results:**

Our review found no standard tool for assessing RoB in prevalence studies of exposure to occupational risk factors. We identified six existing tools for environmental and occupational health systematic reviews and found that their components for assessing RoB differ considerably. With the new RoB-SPEO tool, assessors judge RoB for each of eight domains: (1) bias in selection of participants into the study; (2) bias due to a lack of blinding of study personnel; (3) bias due to exposure misclassification; (4) bias due to incomplete exposure data; (5) bias due to conflict of interest; (6) bias due to selective reporting of exposures; (7) bias due to difference in numerator and denominator; and (8) other bias. The RoB-SPEO's ratings are low, probably low, probably high, high or no information. Pilot testing of the RoB-SPEO tool found substantial inter-rater agreement for six domains (range of *P*_*i*_ for these domains: 0.51–0.80), but poor agreement for two domains (i.e. *P*_*i*_ of 0.31 and 0.33 for biases due to incomplete exposure data and in selection of participants into the study, respectively). Limitations of RoB-SPEO include that it has not yet been fully performance-tested.

**Conclusions:**

We developed the RoB-SPEO tool for assessing RoB in prevalence studies of exposure to occupational risk factors. The tool will be applied and its performance tested in the ongoing systematic reviews for the WHO/ILO Joint Estimates.

## Highlights

• WHO and ILO are systematically reviewing occupational exposure prevalence studies.
• Assessing risk of bias (RoB) in individual studies is a core step of systematic reviews.
• We developed RoB-SPEO, a tool to assess RoB in occupational exposure prevalence studies.
• The performance of RoB-SPEO will be tested in the WHO/ILO systematic reviews.

## 1 Background

The World Health Organization (WHO) and International Labour Organization (ILO) are developing joint estimates of the work-related burden of disease and injury (‘WHO/ILO Joint Estimates’), with contributions from a large network of experts (Ryder, 2017). The organizations will produce global estimates of exposure to selected occupational risk factors (exposure models) and, consecutively, of the burdens of selected diseases and injuries attributable to these exposures (burden of disease models). The organizations are conducting systematic reviews of input data for estimating the burden of 13 pairs of occupational risk factors and health outcomes, whose global burdens of disease have never previously been estimated (Descatha et al., 2018; Hulshof et al., 2019; Paulo et al., 2019; Li et al., 2018; Mandrioli et al., 2018; Godderis et al., 2018; Rugulies et al., 2019; Teixeira et al., 2019; Tenkate et al., 2019).

To parameterize the exposure models, systematic reviews of studies estimating the prevalence (or, in short, prevalence studies) of exposure to occupational risk factors should be performed. WHO and ILO are conducting five such systematic reviews, to synthesize evidence from studies estimating the prevalence of exposure to five diverse occupational risk factors: ergonomic risk factors, dusts and/or fibres, solar ultraviolet radiation, noise, and long working hours. To our knowledge there are no standardized methods for such reviews.

Occupational exposure prevalence studies determine the presence (and often the level) of an exposure to an occupational risk factor in each individual of the study population or in a representative sample at one particular time point (Porta, 2014). They are distinct from studies that estimate incidence, “the number of new health-related events in a defined population within a specified period of time” (Porta, 2014), and prognosis, “the likelihood of future health outcomes in people with a given disease or health condition or with particular characteristics” (p1) (Iorio et al., 2015). Prevalence studies are cross-sectional or longitudinal, whereas incidence studies are always longitudinal. Prevalence studies (as here defined) are purely empirical, whereas prognostic studies are predictive modelling studies (sometimes based on empirical data). Studies estimating prevalence of exposure to occupational risk factors also differ from studies that estimate the effect of an occupational health and safety intervention on a health outcome and those estimating the effect of exposure to an occupational risk factor on a health outcome.

Exposure is the “proximity and/or contact with a source of a disease agent in such a manner that effective transmission of the agent or harmful effects of the agent may occur” (Porta, 2014). Exposures to occupational risk factors are biological, chemical, physical, ergonomic, mechanical and psychosocial exposures among workers at their workplace posing a risk known to be harmful to human health (Ott et al., 2007). One example is workplace exposure to crystalline silica dusts, which are an established physical risk factor for lung cancer among workers (IARC Working Group on the Evaluation of Carcinogenic Risks to Humans, 2009). Both exposure assessment and exposure assignment (the assessment of an exposure based on its determinants including agent, ventilation and worker or environmental characteristic (Burdorf, 2005)) are complex for occupational risk factors. For example, exposures status (whether a worker is exposed or unexposed, or exposed above or below a certain exposure limit) and exposure level (the worker's exposure dose received, expressed in exposure concentration, amount or category) vary within the same worker over time and between different workers in the same occupation, not least because they change as workers' tasks, activities, work processes and work locations change (Burdorf, 2005).

### 1.1 Rationale for development of a new risk of bias tool

**Fig. 1:**
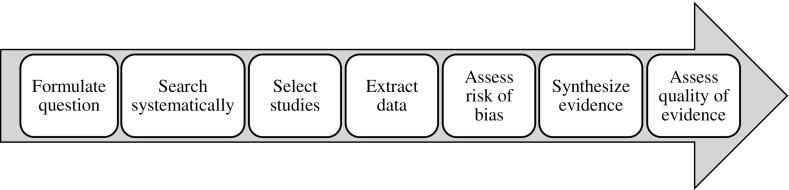
Steps of the systematic review process.

Risk of bias (RoB) is the risk of “a systematic error, or deviation from the truth, in results” (Porta, 2014). Assessing the RoB at the individual study level for each outcome is an essential part of the systematic review process (Fig. 1). Systematic reviews of prevalence studies of exposure to occupational risk factors must therefore also comprise such RoB assessments.

Currently no tool exists for assessing the RoB in prevalence studies of exposure to occupational risk factors (Krauth et al., 2013; Mandrioli and Silbergeld, 2016; Vandenberg et al., 2016; Whaley et al., 2016; Wang et al., 2019), and reviews of parameters for occupational burden of disease studies do not, to our knowledge, encompass such assessments. Of the five methods for assessing the RoB in environmental and occupational health studies identified in a systematic review (Rooney et al., 2016) and a recently published additional instrument (Morgan et al., 2018, Morgan et al., 2019), we consider none applicable for assessing prevalence studies of exposure to occupational risk factors. In fact, no standard and comprehensive RoB assessment method exists for systematic reviews of prevalence studies of exposure in general (Munn et al., 2014). Two checklists for assessing RoB in individual prevalence studies do exist (Hoy et al., 2012; Munn et al., 2014, Munn et al., 2015; The Joanna Briggs Institute, 2017), but RoB assessment should be based on judgment and provide transparent rationales for ratings, and checklists without these features are therefore discouraged.

This lack of a RoB assessment tool for prevalence studies of exposure to occupational (and other) risk factors has implications for occupational (and other) health policy and practice. For example, it challenges evidence-based (occupational) health risk assessments (Table 1 for definitions and steps from International Programme on Chemical Safety, 2004), because health risk assessors do not have a tool available for assessing RoB in the third step: exposure assessment. (The existing tools for assessing RoB in studies of the effect of exposure to occupational risk factors on health outcomes contribute to the first step of health risk assessment, hazard identification, but they cannot be used for the third step, exposure assessment.) Our new tool aims to work towards filling this gap. When formulating the problem for and scoping health risk assessments (National Research Council. Science and Decisions, 2009; World Health Organization, 2010), a RoB assessment tool for prevalence studies could also be useful for identifying options and data needs.

**Table 1:** Definitions of health risk assessment and its steps.

Term/Step	Definition (taken from International Programme on Chemical Safety, 2004)
Health risk assessment	The “process intended to calculate or estimate the risk to a given target organism, system, or (sub)population, including the identification of attendant uncertainties, following exposure to a particular agent, taking into account the inherent characteristics of the agent of concern as well as the characteristics of the specific target system” (p14)
Step 1: Hazard identification	“The identification of the type and nature of adverse effects that an agent has an inherent capacity to cause in an organism, system, or (sub)population” (p13)
Step 2: Hazard characterisation	“The qualitative and, wherever possible, quantitative description of the inherent property of an agent or situation having the potential to cause adverse effects. This should, where possible, include a dose–response assessment and its attendant uncertainties” (p13)
Step 3: Exposure assessment	“Evaluation of the exposure of an organism, system, or (sub)population to an agent (and its derivatives)” (p12)
Step 4: Risk characterisation	“The qualitative and, wherever possible, quantitative determination, including attendant uncertainties, of the probability of occurrence of known and potential adverse effects of an agent in a given organism, system, or (sub)population, under defined exposure conditions” (p14)

Methods for assessing RoB in studies of the effect of exposure to an occupational risk factor on a health outcome (e.g. Office of Health Assessment and Translation, 2015; Lam et al., 2016, and Morgan et al., 2019) cannot directly be applied to assess RoB in occupational exposure prevalence studies for several reasons. Occupational exposure prevalence studies investigate neither health outcomes, nor effects, and consequently any methods in existing tools for assessing RoB related to health outcomes (e.g. RoB in measurement/classification, missing data and selective reporting of health outcomes) and in estimation of effects (e.g. confounding) are not applicable. Some biases (e.g. bias due to differences in numerator and denominator (Williams et al., 2006)) are unique to prevalence studies, and consequently not comprehensively covered in methods in the existing tools for assessing RoB in studies estimating the effect of exposure to occupational risk factors on health outcomes.

Evidence on the effect of an exposure on a health outcome may come from evidence streams other than just human data (here defined as data on exposures among humans collected using personal or other samples), whereas evidence on the prevalence of exposure to an occupational risk factor can come from human data only; methods needed for assessing RoB in studies of effect of exposure to occupational risk factors on health outcomes need to be able to assess RoB across evidence streams, whereas methods for assessing RoB in prevalence studies need to assess human data only. Although components of existing tools for assessing RoB in studies of the effect of exposure to an occupational risk factor on a health outcome are likely applicable (subject to revision, if necessary), existing tools in their entirety cannot simply be applied to assess occupational exposure prevalence studies. We here understand tool components to comprise: instructions (i.e. instructions guiding assessors in their RoB assessments), domains (i.e. distinct domains for assessing defined biases), guiding questions (i.e. questions to prompt assessors), considerations (i.e. specific issues for assessors to consider when assessing defined biases), ratings (i.e. the standard categories for rating RoB) and rating criteria (i.e. the specific criteria for choosing ratings).

Our objective was to develop a valid and reliable tool for assessing the RoB in prevalence studies of exposure to occupational risk factors. The target audience for the tool is researchers who want to assess RoB in studies estimating prevalence of exposures to occupational risk factors among humans. Ideally, a tool should:

• Provide structured and clear guidance to assessors in plain language, including the components of instructions, domains, guiding questions, considerations, ratings and rating criteria.
• Enable comprehensive assessment along domains for all important biases that may present a meaningful risk.
• Enable assessment of any non-randomized study estimating the prevalence of exposure to occupational risk factors in humans.
• Enable differentiated assessment with ratings along defined, clear and unambiguous criteria.
• Enable assessors to document and justify their assessment and rating.

In addition, it is important that this tool be compatible with and complementary to our forthcoming approach for assessing the quality of evidence from a body of prevalence studies of exposure with occupational risk factors, which we are developing in tandem to the tool.

In this paper we describe the development process and present the tool, called the Risk of Bias in Studies estimating Prevalence of Exposure to Occupational risk factors (RoB-SPEO) tool; report results from RoB-SPEO's pilot testing; discuss its potential limitations; and outline suggestions for next steps.

## 2 Methods

### 2.1 Development of the tool

**Fig. 2:**
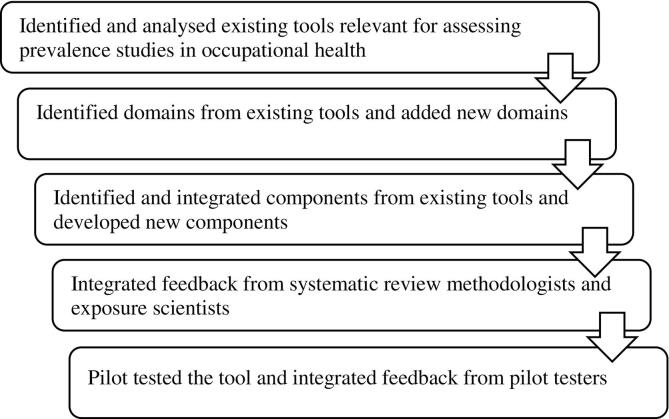
Development of the RoB-SPEO tool.

WHO and ILO, collaborating with a large network of systematic review methodologists and experts of occupational and environmental health and exposure science, have developed RoB-SPEO specifically for assessing RoB in individual studies estimating the prevalence of exposure to occupational risk factors. The steps for development of the tool are summarized in Fig. 2, and each step is described in more detail below.

#### 2.1.1 Identified and analysed existing tools relevant for assessing prevalence studies in occupational health

We undertook a comprehensive, but non-systematic review of the literature in the electronic academic databases Ovid Medline and EMBASE in June 2018 to identify and select existing RoB tools that might be relevant to assessing studies estimating the prevalence of exposure to occupational risk factors. Tools were considered relevant if they focus on prevalence studies; incidence studies; prognostic studies; studies on the effect of exposure to occupational risk factors on health outcomes; and/or studies of the effect of occupational health and safety interventions on health outcomes. We focused specifically on the tools (or tool components) for assessing RoB in non-randomized studies of human data, because prevalence studies of interest to us are of this type. Tools (or tool components) for randomized study designs and in vitro, in vivo and/or mechanistic evidence streams and instruments other than RoB tools (e.g. reporting guidelines) were excluded and not further considered. Thirty experts in systematic review methods, occupational health and occupational exposure science contributing to the systematic reviews for the WHO/ILO Joint Estimates were also asked to identify tools.

#### 2.1.2 Identified domains from existing tools and added new domains

We decided on eligibility criteria for including domains from existing tools in RoB-SPEO. We included domains, if they were relevant (i.e. applicable directly or after modification) for any of:

• Non-randomized studies.
• Studies estimating any prevalence.
• Studies estimating any incidence.
• Studies on human data.
• Assessment of exposure.

Domains were excluded if they were exclusively relevant for:

• Randomized studies.
• Statistical or mathematical modelling studies.
• Studies estimating the effect of an intervention.
• Studies estimating the effect of an occupational or other exposure.
• Studies on in vitro, in vivo, animal and/or mechanistic data.
• Assessment of a health outcome.
• Comparison to an ideal target intervention or exposure.

We applied these eligibility criteria to select RoB domains of relevance for RoB-SPEO. We tailored domains designed for outcomes data (e.g. missing *outcomes* data and selective *outcomes* reporting) into domains for exposure data in the RoB-SPEO tool (e.g. incomplete *exposure* data and selective *exposure* reporting). We also identified new domains that were crucial for RoB-SPEO but did not appear in any of the existing tools.

#### 2.1.3 Identified and integrated components from existing tools and developed new components

We screened the components of all existing tools identified in our search for their applicability for the RoB-SPEO. We applied the same criteria that we used to determine applicability of domains also to determine applicability of individual components. Subsequently, we integrated applicable components under the relevant RoB-SPEO domain or domains. Components were either adopted verbatim (rarely) or modified (often substantially) to suit the RoB-SPEO (commonly). Finally, for RoB-SPEO domains without any (or without core) components identified as applicable from existing tools, we developed these components.

The Navigation Guide systematic review framework comprises a RoB tool that was developed in 2011 based on tools from Cochrane (Higgins and Green, 2011) and the Agency for Healthcare Research and Quality (Viswanathan et al., 2008). It is tailored specifically to systematic reviews of occupational health studies and includes components for assessing RoB in exposure assessment and exposure assignment (i.e. the assessment of an exposure based on its determinants (Burdorf, 2005)). We considered it to be the perhaps most relevant tool for our RoB-SPEO tool development. Consequently, if components were shared by the Navigation Guide and other existing tools, we departed from the components of the latest version (Lam et al., 2016) of the Navigation Guide tool.

We could perhaps have similarly also departed primarily from the RoB instrument for non-randomized studies of exposures (Morgan et al., 2019), but this tool is new and perhaps less straightforward. Using the Navigation Guide tool as a common departure point for component development (where feasible) was also seen as helping us ensure compatibility of RoB-SPEO with existing systematic review frameworks, including the Navigation Guide one. Moreover, for the development of the WHO/ILO Joint Estimates, systematic reviews of the effect of exposure to occupational risk factors on health outcomes are using the Navigation Guide RoB tool (Descatha et al., 2018; Hulshof et al., 2019; Paulo et al., 2019; Li et al., 2018; Mandrioli et al., 2018; Godderis et al., 2018; Rugulies et al., 2019; Teixeira et al., 2019; Tenkate et al., 2019), and we sought parity in RoB assessment methods with the systematic reviews of studies estimating prevalence of exposure to occupational risk factors, in as much as is possible.

A good example of the process we underwent in component development is that for the development of the standard ratings for RoB-SPEO: First, we screened the existing tools and identified the ratings they used. Second, as we found ratings of the Navigation Guide to be potentially applicable for RoB-SPEO, we departed from that tool and adopted four of its ratings: “low risk”, “probably low risk”, “probably high risk” and “high risk”. Third, we decided to not include the Navigation Guide's fifth rating (“not applicable”), as we considered assessment of all eight RoB domains is applicable to all eligible prevalence studies. This is consistent with the United States Office for Health Assessment and Technology (OHAT) tool (*Office of Health Assessment and Translation*, 2015). Finally, we decided that a rating category was needed that enabled assessors to indicate that due to poor reporting they could not rate RoB. We therefore adopted the rating “no information” from the ROBINS-I tool (Sterne et al., 2016) and the RoB instrument for non-randomized studies of exposures (Morgan et al., 2019) as RoB-SPEO's fifth (and final) rating.

Financial and other interests could introduce bias in prevalence studies of exposure to occupational risk factors, depending on interests leading to underestimation, overestimation and selective reporting of exposures known to harm human health at workplaces. We departed from the components of the conflict of interest domain from the Navigation Guide but revised them substantially to align with the standards established in the International Committee of Medical Journal Editors (ICMJE) *Form for Disclosure of Potential Conflicts of Interest* (International Committee of Medical Journal Editors, n.d). ICMJE identifies four categories of potential real or perceived conflicts of interest (see Appendix 1 in Supplementary data) in relation to work presented in published study records, relevant financial activities outside the published study record, intellectual property and other relationships. Non-financial conflicts of interest are not always consistently and comprehensively defined and reported despite the established ICMJE standards, and some experts do not view them as carrying risk of introducing bias (Bero and Grundy, 2016; Bero, 2017).

We recognize that there are several, substantial differences between the RoB-SPEO tool and existing domain-based RoB assessment tools. Compared with the Navigation Guide and OHAT tools for example, RoB-SPEO is for assessing prevalence studies, not studies of the effect of an exposure on a health outcome (or hazardousness or toxicity of an exposure to human health). Consequently, RoB-SPEO necessarily comprises several components that do not appear in the existing tools. We therefore developed several new components for RoB-SPEO specifically. For example, the inclusion of a domain for assessing RoB due to differences in numerator and denominator (sometimes called numerator-denominator bias (Williams et al., 2006)) is unique and specific to prevalence studies, and we therefore developed all components for this new domain in our tool.

#### 2.1.4 Integrated feedback from systematic review methodologists and exposure scientists

We sought and integrated feedback on RoB-SPEO in five stages (Table 2). In total, feedback on RoB-SPEO's content, structure and formatting was gathered from:

• Nine WHO and ILO experts.
• One hundred and thirty individual experts who contribute to the ongoing WHO/ILO systematic reviews, including systematic review methodologists and occupational health, occupational safety and exposure scientists.
• Ten experts in systematic review methods external to the WHO/ILO systematic reviews, including members of Cochrane, the Navigation Guide Working Group and the GRADE Environmental Health Project Group.
• Nine pilot testers of the tool.
• Twenty external peer reviewers and journal editors.

**Table 2:** Stages of receipt and integration of feedback during the development of RoB-SPEO and main innovations introduced.

Stage	Feedback receipt and integration	Main innovations introduced
1	Three rounds of feedback on RoB-SPEO version (v.) 1 received from seven WHO and ILO experts and 130 individual experts on systematic review methods, occupational health and safety and/or exposure science and integrated in RoB-SPEO v.2	- Adopted and/or revised tool components from existing tools, including instructions, domains, guiding questions, considerations, ratings and rating criteria - Introduced concepts, terms and examples from occupational health and safety
2	Feedback on RoB-SPEO v.2 received from five external systematic review methodologists, 20 peer reviewers and two journal editors received, integrated in RoB-SPEO v.3, and this tool version published in systematic review protocols (e.g. Mandrioli et al., 2018) as prototype	- Further refined tool components, especially rating criteria - For each domain, added a description of the bias covered
3	Three rounds of feedback on RoB-SPEO v.3 received from eight WHO and ILO experts and 30 individual experts in systematic review methods, occupational health, occupational safety and/or exposure science and integrated in RoB-SPEO v.4	- Revised instructions - Substantially revised guiding questions - To improve comprehension and specificity, added several examples from exposure science - Further refined rating criteria - For each domain, added essential definitions of key terms and concepts
4	One round of feedback on RoB-SPEO v.4 received from pilot testers and integrated in RoB-SPEO v.5	- Introduced additional domain on bias due to differences in numerator and denominator - Introduced standard tables for reporting assessments by domain - Introduced new rating category (“No information”)
5	Feedback on RoB-SPEO v.5 received from two external systematic review methodologists, four peer reviewers and a journal editor and integrated in RoB-SPEO v.6 presented in this article	- Re-formatted tool for clarity, introducing subheadings to differentiate components - In the tables for reporting assessments by domain, added reporting of data extracted from study records to support assessments and ratings

This extensive expert feedback was used to sequentially develop the tool, starting from an initial prototype (RoB-SPEO version (v.) 1) and finally arriving at the tool presented in this article (RoB-SPEO v.6; Appendix 1 in the Supplementary data). Selected, main innovations introduced at each stage are presented in Table 2.

#### 2.1.5 Pilot tested tool and integrated feedback from pilot testers

We pilot-tested the RoB-SPEO tool, calculated inter-rater agreement, and analysed and integrated feedback received from pilot testers to further improve the tool. First, for each ongoing WHO/ILO systematic review of prevalence studies (Descatha et al., 2018; Hulshof et al., 2019; Paulo et al., 2019; Li et al., 2018; Mandrioli et al., 2018; Godderis et al., 2018; Rugulies et al., 2019; Teixeira et al., 2019; Tenkate et al., 2019), we extracted from the Endnote library for the systematic review a list of all included study records; ordered the records alphabetically by surname of the first author; and numbered them starting at 1 (sampling frame). For each systematic review, we generated one random number between 1 and the highest number allocated to any study record for the systematic review using an online tool (https://www.random.org/) and then selected the study record with this number for pilot tests. This ensured pilot testing on a sample of five randomly drawn study records of prevalence studies of exposure with ergonomic, physical, chemical and psychosocial occupational risk factors (Cattaneo et al., 2012; Gies et al., 2009; Strauss et al., 2014; Kim et al., 2016; Hartling et al., 2013).

Second, nine co-authors of this article pilot tested RoB-SPEO version (v.) 4. This included three experts who had not previously contributed to RoB-SPEO's development. Each pilot tester was allocated two study records for assessment. We asked the pilot testers to conduct their assessments using RoB-SPEO v.4; record the outcomes (i.e., ratings and justifications) of the assessments; propose revisions to the tool to address identified issues; and, if unable to address an issue, record this issue.

Third, to preliminarily test RoB-SPEO's performance and to identify domains achieving poor agreement for further development, we calculated a raw measure of agreement in ratings between pilot testers by domain. There is little scientific consensus on which of several existing methods is preferred for calculating such inter-rater agreement. We extracted ratings by pilot tester, study record and domain. The six ratings used by pilot testers were coded into three analytical categories: (1) “Low/Probably low”, (2) “High/Probably high” and (3) “Unclear/Cannot be determined”. We applied established methods (Morgan et al., 2018; Armijo-Olivo et al., 2012; Bilandzic et al., 2016; Savovic et al., 2014; Losilla et al., 2018) to calculated the proportion of all ratings given by all pilot testers to the *j-th* analytical category (*P*_*i*_), using the following formula:

$$P_{i}=\frac{1}{n\left(n-1\right)}\sum_{j=1}^{k}n_{\mathrm{ij}}\left(n_{\mathrm{ij}}-1\right)$$

where *i = 1,…k* is the number of domains (here, k = 7); *j = 1,…k* is the number of possible analytical categories (here, k = 3); and *n* = number of assessors for the study record. P_i_ ranges from 0.00 (no two pilot testers chose the same rating) to 1.00 (all pilot testers chose the same rating). We defined values of *P*_*i*_ > 0.80 as “Almost perfect agreement”; 0.4 ≤ *P*_*i*_ ≥ 0.8 as “Substantial agreement”; and *P*_*i*_ < 0.4 as “Poor agreement”. However, we note that inter-rater agreement measures are not necessarily indicators for tool performance, since ratings are explicitly based on individual judgment (Higgins et al., 2011).

Finally, we integrated any feedback received from pilot testers, including proposals for revisions to RoB-SPEO v.4 and identifications of issues for further tool development, to develop RoB-SPEO v.5. Our analysis of inter-rater agreement enabled us to especially focus the developments of RoB-SPEO versions 5 and 6 on domains identified as having only poor inter-rater agreement in RoB-SPEO v.4.

## 3 Results

### 3.1 Existing tools relevant for RoB-SPEO

We identified six existing tools for assessing RoB that we considered most relevant (Table 3), including four domain-based tools: the Risk Of Bias In Non-randomized Studies of Interventions (ROBINS-I) tool (Sterne et al., 2016), the RoB instrument for non-randomized studies of exposures (Morgan et al., 2019), the Navigation Guide's (Lam et al., 2016) and the United States Office of Health Assessment and Translation's (OHAT's) (*Office of Health Assessment and Translation*, 2015). We excluded several tools and other instruments from our review (e.g. Higgins et al., 2018; Sterne et al., 2014; Wells et al., 2019; Stevens et al., 2016; The GATHER Working Group, 2016; ), because they were designed specifically for assessing irrelevant study types and/or were instruments other than RoB tools (see Appendix 2 in the Supplementary data for selected excluded tools and the rationale for their exclusion). The relevant existing tools assess RoB in studies of the effect of interventions (including occupational health and safety ones) and the health effect (or hazardousness or toxicity) of exposure to environmental and occupational risk factors. No domain-based tool for prevalence studies was identified, however.

**Table 3:** Existing risk of bias tools identified as relevant for the development of RoB-SPEO.

	Tool	Study types assessed	Why this tool is not applicable
	*Domain-based tools*		
1	ROBINS-I tool (Sterne et al., 2016)	Non-randomized studies of the effect of interventions on health outcomes	- Has components applicable exclusively to health outcomes - Has components applicable exclusively to intervention effectiveness - Is not tailored to studies estimating prevalence - Is not tailored to occupational health, occupational safety and exposure scientific studies
2	RoB instrument for non-randomized studies of exposures (Morgan et al., 2019)	Non-randomized studies of the effect of exposure to environmental and occupational risk factors on health outcomes	- Has domains applicable exclusively to health outcomes - Has domains applicable exclusively to studies of the effect of exposure to environmental and occupational risk factors on health outcomes - Is not tailored to studies estimating prevalence
3	Navigation Guide RoB tool (Lam et al., 2016)		
4	OHAT RoB tool (*Office of Health Assessment and Translation*, 2015)		
	*Checklists*		
5	Hoy et al. RoB checklist (Hoy et al., 2012)	Prevalence studies of health outcomes	- Uses a checklist approach - Is tailored to neither occupational health and safety nor exposure scientific studies
6	Munn et al. critical appraisal checklist (Munn et al., 2014; Munn et al., 2015; The Joanna Briggs Institute, 2017)		

Two checklists (Munn et al., 2014; Hoy et al., 2012) for assessing RoB in prevalence studies of health outcomes were identified (Table 3). Assessing RoB using checklists is not consistent with latest methods for RoB assessment, which are based on judgment (Bero, 2014). In addition, these checklists were tailored to neither exposure science, nor occupational health and safety, nor a related field (e.g. environmental health). Nevertheless, the checklists did add important considerations for the new tool; for example, they were the only tools that provided considerations for assessing bias due to differences in numerator and denominator (Williams et al., 2006).

Some additional tools were examined. The GATHER Working Group outlines selected RoB categories for reporting input data, which often include prevalence estimates of exposure to risk factors affecting human health. We also considered our forthcoming approach for assessing quality of evidence in occupational risk factor prevalence studies, because we wanted to develop a RoB tool that would be useful for and compatible with this approach.

We considered none of the existing tools to be applicable to assessing RoB in studies estimating the prevalence of exposure to occupational risk factors (Table 3 for details). The existing domain-based tools are developed to assess other types of studies and therefore comprise domains and other components that are exclusively applicable to these studies, and one of the domain-based tools is in additional also tailored neither to occupational health, nor to occupational safety, nor to occupational exposure scientific studies. Checklist approaches are increasingly abandoned (Bero, 2014), and existing checklists are also tailored neither to occupational (or environmental) health, nor to occupational (or other) exposure scientific studies.

### 3.2 Domains relevant for RoB-SPEO

From the four domain-based tools that resulted relevant to our scope in our search (Table 3), we identified and selected five domains (Table 4). We added an additional three domains that were not included in existing tools, but we considered essential for RoB-SPEO (Table 4). The rationale for selecting domains on these biases was that we considered these biases to be of potentially meaningful size in prevalence studies of exposure to occupational risk factors, as well as applicable for assessing RoB in these studies. In contrast, as per our exclusion criteria, we excluded domains from existing tools that assessed bias due to confounding; deviations from intended interventions or exposures; and classification of interventions, as well as bias in classification of interventions and measurement of outcomes, respectively (Appendix 3 in the supplementary data for rationales for the exclusion of domains from existing tools). If necessary, we modified the names of domains to ensure their full fit to the new tool; for example, we changed the domain called “Incomplete outcome data” to “Bias due to incomplete exposure data” and “Selective outcome reporting” to “Bias due to selective reporting of exposures”. To improve user friendliness and comprehension, as has been done in the revised Cochrane RoB 2.0 (Sterne et al., 2014) and ROBINS-I (Sterne et al., 2016) tools, we moved towards using more explicit bias names (e.g. “Bias due to incomplete exposure data”, rather than just “Incomplete exposure data”). The thirty experts in systematic review methods, occupational health, occupational safety and exposure science contributing to the systematic reviews for the WHO/ILO Joint Estimates, including the authors of this article, and selected additional experts (see Acknowledgments section) were also asked to identify missing domains, but identified none. The ordering of the domains in RoB-SPEO is consistent with the ordering of the same (or similar) domains in existing RoB tools, which should facilitate the use of the tool among its users and contribute to the harmonization of domain-based tools.

**Table 4:** Domains of risk of bias in RoB-SPEO.

	Domain	Description of bias	Existing tools with such a domain
1	Bias in selection of participants into the study	Bias in selection of participants into the study (commonly called selection bias) is the bias due to systematic differences between the characteristics of the study sample (defined as the sample of individuals participating in the study) and those of the target population (defined as the population for which the authors of the study sought to assess exposure) (Porta, 2014).	ROBINS-I, Risk of bias (RoB) instrument for non-randomized studies of exposures, Navigation Guide RoB tool, OHAT RoB tool
2	Bias due to a lack of blinding of study personnel	Bias due to a lack of blinding of study personnel (commonly called performance bias) is the bias that arises due to a lack of blinding of exposure assessors and other study personnel to relevant participant characteristics (e.g. disease status) that leads to exposure assessment that differs depending on participant characteristics.	Navigation Guide RoB tool, OHAT RoB tool
3	Bias due to exposure misclassification	Bias due to exposure misclassification is “erroneous [and systematic] classification of an individual, a value, or an attribute into a [exposure] category other than that to which it should be assigned”, leading to under- or over estimation of prevalence of exposure status (or level) (Porta, 2014).	ROBINS-I, RoB instrument for non-randomized studies of exposures, Navigation Guide RoB tool, OHAT RoB tool
4	Bias due to incomplete exposure data	Bias due to incomplete exposure data is the biases that arises from exposure data missing in a way that the exposure assessment is differential by exposure status (or level) in the target population (i.e. not random).	None, but existing tools do have domains on bias due to missing data in general or missing outcome data
5	Bias due to selective reporting of exposures	Bias due to selective exposure reporting is the systematic difference arising from selective reporting of exposures or exposure categories.	None, but existing tools do have domains on bias due to selective reporting in general or selective reporting of outcomes
6	Bias due to conflict of interest	Bias due to conflicts of interest is the bias introduced if financial and other interests influence the design, conduct, data collection, analysis and/or reporting of a study (Woodruff and Sutton, 2014).	Navigation Guide RoB tool
7	Bias due to differences in numerator and denominator	Bias due to differences in numerator and denominator is the bias that arises when there is a mismatch of definition and/or counting of persons contributing to the numerator and the denominator in the ratio used to estimate prevalence (Williams et al., 2006).	None
8	Other bias	Other bias is any other bias specific to a particular study rather than applicable to all studies.	ROBINS-I, RoB instrument for non-randomized studies of exposures, Navigation Guide RoB tool, OHAT RoB tool

### 3.3 Description of RoB-SPEO

RoB-SPEO, the new tool for assessing RoB in studies estimating the prevalence of exposure to occupational risk factors, is presented in full in Appendix 1 in the Supplementary data. RoB-SPEO's overall structure comprises first general instructions and then the eight RoB domain sections (one section per domain). This structure is similar to that of both the Navigation Guide (Lam et al., 2016) and the OHAT (*Office of Health Assessment and Translation*, 2015) tools. This similarity may perhaps aid conjoint use of these existing tools and our new tool, and it may also contribute to establishing agreed standard structures across RoB tools.

#### 3.3.1 General instructions

RoB-SPEO provides general instructions to assessors upfront to guide their assessments, and these include:

• Choose one of four ratings (i.e., low, probably low, probably high or high).
• Choose the category “no information”, if the information required to rate RoB is not reported or reported too incompletely.
• Avoid double-counting of risks of bias across domains.
• Always extract from the study record(s) the data that your judgment is based on.
• Always provide a detailed justification for each judgment for each domain.

#### 3.3.2 Eight risk of bias domain sections

All RoB-SPEO's eight RoB domains sections comprise the same five components in the same order:

1. Guiding question.
2. Description of bias and/or definitions of key terms and concepts
3. Considerations.
4. Ratings and rating criteria.
5. Table for recording the assessment.

##### 3.3.2.1 Guiding questions

For each of the eight domains, the tool provides a guiding (or signalling) question as prompt for the assessor (Table 5).

**Table 5:** Guiding questions by domain.

	Domain	Guiding question
1	Bias in selection of participants into the study (see Table 4 for description of biases)	Could the exposure status (or level) assessed (or assigned) in the study sample not represent exposure in the target population?
2	Bias due to a lack of blinding of study personnel	Could study personnel have known the exposure status (or level) or other characteristics of study participants and, if yes, could this knowledge have influenced how they conducted the exposure assessment?
3	Bias due to exposure misclassification	Could the methods used for assessing (or assigning) exposure have over- or under-estimated exposure?
4	Bias due to incomplete exposure data	Could data on exposure status (or level) be incomplete for eligible participants?
5	Bias due to selective reporting of exposures	Could relevant exposures or exposure categories be selectively not reported?
6	Bias due to conflict of interest	Could the study and/or one or more study authors have received support from entities with potential interests in the exposure assessed (or assigned)?
7	Bias due to differences in numerator and denominator	Could the definition and/or counting of persons contributing to the numerator differ from those contributing to the denominator in the ratio used to estimate prevalence?
8	Other bias	Could the study have other problems that could have introduced bias?

##### 3.3.2.2 Description of bias and/or definitions of key terms and concepts

The guiding question is followed by a description of the specific bias to be assessed. These are the descriptions presented above in Table 4. We added these descriptions to ensure that assessors have a shared understanding of the bias for which they are to assess risk.

For some domains, the concept of bias in studies estimating prevalence differs substantially from that for bias in studies estimating effects. For example, bias in prevalence does not necessarily have to be differential to matter (e.g. non-differential misclassification leads to bias in prevalence estimates in the form of under- or overestimation). Bias in effect estimates on the other hand often matters most if it is differential (e.g. whereas non-differential misclassification can introduce bias in effect estimates, this bias is nearly always towards the null, and it is differential misclassification that can result in bias in either direction). This has substantial implications for the description of bias, as well as across components, in RoB-SPEO, and we have tailored the tool's component accordingly (especially considerations and rating criteria). Additional refinements of RoB-SPEO are probably needed to fully address differential versus non-differential bias in assessments with this tool.

Where relevant, we identified key terms and concept that we considered central to understanding components of section for this RoB domain, but for which assessors may not have a shared understanding. The tool presents definitions of these central terms and/or concepts, to ensure terminological and conceptual understanding and clarity across assessors.

##### 3.3.2.3 Considerations

For most RoB domains, a set of key considerations is presented. These considerations are non-exhaustive, and some considerations apply to selected study designs or exposure assessments (or assignment) methods only. Nevertheless, they seek to prompt and guide assessors to consider potentially relevant issues when conducting their assessment for a RoB domain.

##### 3.3.2.4 Ratings and rating criteria

RoB-SPEO enables assessment along five ratings of RoB as:

1. Low.
2. Probably low.
3. Probably high.
4. High.
5. No information.

Separately for each rating, staring from “low” and finishing at “high”, the tool presents criteria for each rating. The rating “no information” enables assessors to indicate that the information required to rate RoB is either not reported at all, or reported too incompletely to rate RoB with sufficient confidence.

##### 3.3.2.5 Table for recording the assessment

**Fig. 3:**
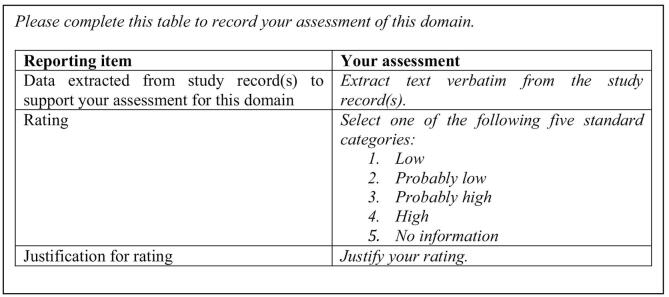
Instructions (in Italics) and table for recording the assessment.

The final component of the section for assessing each RoB domain is a table that enables assessors to record their assessments (Fig. 3). Assessors record their rating, the justification for this rating and the data extracted from the study record to support the rating and its justification.

### 3.4 Interpretation and summary of overall risk of bias across domains

Table 6 presents the interpretation of overall RoB ratings in an individual study across domains in RoB-SPEO. The overall RoB for a study is the rating given in any of the eight domains that indicates the highest RoB. So, for example, if assessors assessed the RoB in selection of participants into the study as being “Probably high” and the risk of all other biases as “Low”, then the overall RoB for the study is “Probably high”. As in the Navigation Guide systematic review framework (Johnson et al., 2014), RoB heat maps are used as a visual summary of RoB across domains for individual studies (for an example see Figure 3 on p1033 in da Costa et al., 2017).

**Table 6:** Interpretation of overall risk of bias ratings in RoB-SPEO.

Rating (or judgment)	Interpretation
Low risk of bias	The study is judged to be at low risk of bias for all domains
Probably low risk of bias	The study is judged to be at low or probably low risk of bias for all domains
Probably high risk of bias	The study is judged to be at probably high risk of bias in at least one domain, but not at high risk of bias in any domain
High risk of bias	The study is judged to be at high risk of bias in at least one domain

Footnotes: Ratings are adapted and interpretations adopted verbatim from the ROBINS-I tool; see Table 2 in Bero, 2014.

### 3.5 Agreement in ratings among pilot testers

Using a raw measure of agreement (*P*_*i*_) between pilot testers for the RoB-SPEO v.4, this tool version achieved “substantial agreement” (0.40 ≤ *P*_*i*_ ≤ 0.80) for six domains:

• Bias due to conflict of interest: 0.51 (*P*_*i*_).
• Other bias: 0.51.
• Bias due to a lack of blinding of study personnel: 0.65.
• Bias due to exposure misclassification: 0.76.
• Bias due to selective reporting of exposures: 0.80.

However, we found “poor agreement” (*P*_*i*_ < 0.40) for two domains:

• Bias due to incomplete exposure data (0.31)
• Bias in selection of participants into the study (0.33).

The poor levels of agreement for the two domains can be at least partially explained by the fact that normally RoB is independently performed by two trained assessors and then consensus is reached among assessors, increasing the level of agreement (Fleiss, 1971). In fact, in general agreement among RoB assessors should be calculated among pairs of assessors, not single assessors. Different numbers of pilot testers assessed each study, so Fleiss kappa (Fleiss, 1971) could not be calculated; considering the small number of articles assessed, Fleiss kappa would however not have been very informative.

## 4 Discussion

### 4.1 Comparison of RoB-SPEO with selected previous tools

Table 7 presents a comparison of RoB-SPEO with the four existing, relevant domain-based tools. The main differences between RoB-SPEO and the ROBINS-I tool; the RoB instrument for non-randomized studies of exposures; the Navigation Guide tool; and the OHAT tools are:

• RoB-SPEO seeks to assess RoB in prevalence studies of the exposure to occupational risk factors. The other tools seek to assess RoB in studies of the causal effect (or association) on health outcomes of either interventions or of exposure to occupational or environmental risk factors.
• RoB-SPEO, the Navigation Guide and the OHAT tools do not use a target study approach, whereas the ROBINS-I tool and the RoB instrument for non-randomized studies of exposures do.
• RoB-SPEO only assesses RoB along domains relevant to non-randomized prevalence studies of humans, whereas the other tools assess along domains relevant to health outcomes (e.g. outcome misclassification bias, missing outcomes data and selective outcomes reporting); effects of interventions on health outcomes (e.g. confounding); and effects of exposure with environmental and occupational risk factors on health outcomes (e.g. confounding).
• RoB-SPEO adopts with minor modifications the four standard ratings of the Navigation Guide and OHAT tools, whereas the ROBINS-I tool uses a different set of ratings and the Navigation Guide adds a fifth rating (“Not applicable”).
• RoB-SPEO, the Navigation Guide and the OHAT tools have one guiding question for each domain (see footnotes in Table 7), whereas the ROBINS-I tool does not have one, but several, signalling question per domain.

**Table 7:** Comparison of existing tools and RoB-SPEO.

Tool	Types of studies	Evidence streams	Domains	Ratings (or judgments)
ROBINS-I (Sterne et al., 2016)	Non-randomized studies of the effect of interventions on health	Human data	Pre-intervention: 1. Bias due to confounding 2. Bias in selection of participants into the study At intervention: 3. Bias in classification of interventions Post-intervention: 4. Bias due to deviations from intended interventions 5. Bias due to missing data 6. Bias in measurement of outcomes 7. Bias in selection of the reported result	1. Low risk of bias 2. Moderate risk of bias 3. Serious risk of bias 4. Critical risk of bias 5. No information
Risk of bias (RoB) instrument for non-randomized studies of exposures (Morgan et al., 2019)	Studies of the effect of exposure to environmental and occupational risk factors on health outcomes (in the context of health risk assessment: hazard identification; see Table 1)	Human data	1. Bias due to confounding 2. Bias in selection of participants into the study 3. Bias in misclassification of exposure 4. Bias due to deviations from intended exposures 5. Bias due to missing data 6. Bias in measurement of outcomes 7. Bias in selection of the reported result	1. Low risk of bias 2. Moderate risk of bias 3. Serious risk of bias 4. Critical risk of bias 5. No information
Navigation Guide RoB tool (human evidence stream) (Lam et al., 2016)	Studies of the effect of exposure to environmental and occupational risk factors on health outcomes (hazard identification)	In vitro, animal, mechanistic and human data	1. Selection bias ^a^ 2. Performance bias ^b^ 3. Exposure misclassification bias ^c^ 4. Outcome misclassification bias ^d^ 5. Confounding ^e^ 6. Incomplete outcome data ^f^ 7. Selective outcome reporting ^g^ 8. Conflict of interest ^h^ 9. Other bias ^i^	1. Low risk 2. Probably low risk 3. Probably high risk 4. High risk 5. Not applicable
OHAT RoB tool (cohort, case-control, cross-sectional and case series studies in human evidence stream) (Office of Health Assessment and Translation, 2015)	Studies of the effect of chemicals on health outcomes (hazard identification)	In vitro, animal, mechanistic and human data	1. Selection bias ^j^ 2. Confounding bias ^k^ 3. Performance 4. Attrition/Exclusion bias ^l^ 5. Detection bias ^m^ 6. Selective reporting bias ^n^ 7. Other ^o^	1. Definitely low risk 2. Probably low risk 3. Probably high risk 4. Definitely high risk
RoB-SPEO (see Appendix 1 in the Supplementary data)	Prevalence studies of the exposure to occupational risk factors (in the context of health risk assessment: exposure assessment)	Human data	1. Bias in selection of participants into the study 2. Bias due to a lack of blinding of study personnel 3. Bias due to exposure misclassification 4. Bias due to incomplete exposure data 5. Bias in selection of the reported results 6. Bias due to conflict of interest 7. Bias due to differences in numerator and denominator 8. Other bias	1. Low risk 2. Probably low risk 3. Probably high risk 4. High risk 5. No information

Footnotes: Guiding questions are as follows: ^a^ Are the study groups at risk of not representing their source populations in a manner that might introduce selection bias? ^b^ Was knowledge of the group assignments inadequately prevented (i.e., blinded or masked) during the study, potentially leading to subjective measurement of either exposure or outcome? ^c^ Were exposure assessment methods lacking accuracy? ^d^ Were outcome assessment methods lacking accuracy? ^e^ Was potential confounding inadequately incorporated? ^f^ Were incomplete outcome data inadequately addressed? ^g^ Does the study report appear to have selective outcome reporting? ^h^ Did the study receive any support from a company, study author, or other entity having a financial interest in any of the exposures studied? ^i^ Did the study appear to have other problems that could put it at a risk of bias? ^j^ Did selection of study participants result in appropriate comparison groups? ^k^ Did the study design or analysis account for important confounding and modifying variables? ^l^ Were outcome data complete without attrition or exclusion from analysis? ^m^ Can we be confident in the exposure characterisation? and Can we be confident in the outcome assessment? ^n^ Were all measured outcomes reported? ^o^ Were there no other potential threats to internal validity (e.g., statistical methods were appropriate and researchers adhered to the study protocol)?

### 4.2 RoB-SPEO's strengths and limitations

A major strength of RoB-SPEO is that its development was based on extensive and repeated rounds of feedback from many diverse experts on systematic review methods, occupational health and safety, environmental health and exposure science. RoB-SPEO has also already been pilot tested, and the preliminary results from the pilot tests suggest that RoB-SPEO performs well across most domains, achieving an overall good level of inter-rater agreement.

Limitations of RoB-SPEO include that our review of existing tools was non-systematic, when it should ideally have been systematic; however, we asked several experts to identify existing tools for assessing RoB in studies estimating prevalence of exposure to occupational risk factors, and they did not identify any existing domain-based tools, and this increases our confidence in the conclusion that no prior such tool exists. RoB-SPEO may not currently comprehensively enough guide assessments of risk of differential versus non-differential biases. Another limitation is that further studies are needed to performance-test the tool and its different components (see next section).

### 4.3 Further tool testing and development

RoB-SPEO has face validity and has been pilot-tested, but its performance requires further testing. This crucially includes comprehensive assessment of RoB-SPEO's inter-rater reliability. We acknowledge that inter-rater reliability cannot establish epistemological reliability (i.e. the reliability of the methods used to assess RoB) and that a tool based on expert judgment cannot necessarily be expected to be reliable and should not be assessed against quantitative reliability alone or even primarily. Considering that pilot testing of RoB-SPEO v.4 found poor inter-rater agreement for domains 1 (bias in selection of participants into the study) and 4 (bias due to incomplete exposure data), these two domains require additional and especially stringent testing when RoB-SPEO v.6 is performance-tested. To clarify the potential limits of the tool, evaluations are needed of agreement among pairs of assessors, rather than single assessors, on a large number of studies using suitable metrics (e.g. Fleiss' kappa (Fleiss, 1971)). The application of RoB-SPEO in all ongoing systematic reviews of prevalence studies of exposure with occupational risk factors that are currently being conducted for the WHO/ILO Joint Estimates (Descatha et al., 2018; Hulshof et al., 2019; Paulo et al., 2019; Li et al., 2018; Mandrioli et al., 2018; Godderis et al., 2018; Rugulies et al., 2019; Teixeira et al., 2019; Tenkate et al., 2019) will provide an opportunity for quantitative testing of our new tool's performance, as part of the WHO/ILO Work-Related Burden of Disease and Injury Study.

We have identified some areas for potential further consideration as the tool is being further developed. Some experts expressed concern that some domains overlap risking double-counting of RoB across these domains, including the domains on:

• Bias in selection of participants into the study and bias due to exposure misclassification.
• Bias in selection of participants into the study and bias due to incomplete exposure data.
• Bias due to a lack of blinding of study personnel and bias due to exposure misclassification.

Collapsing domains should therefore be further considered. The ordering of domains could also be further considered and tested. Assessing RoB from (especially non-financial) conflict of interest is still novel, and consequently components of this domain may require further refining in the future. RoB-SPEO probably needs further refinement to more comprehensively address assessments of risk of differential versus non-differential biases. Potentially within the scope of the tool are also studies that use mathematical or statistical modelling of empirical exposure measurement and studies using biomarkers for measuring exposure, but it is probably warranted to develop dedicated considerations and rating criteria for each relevant domain to assess RoB in these studies.

RoB-SPEO will benefit from further development and refinement over time, if and when gaps in or problems with it are found. We welcome feedback from RoB-SPEO users, not least from exposure scientists, to further tailor and refine tool components, especially for domains relevant to exposure assessment (and/or assignment).

## 5 Conclusions

We have developed RoB-SPEO, a tool for assessing RoB in primary studies estimating prevalence of exposure to occupational risk factors. RoB-SPEO can potentially be applied in systematic reviews for health risk assessment (i.e. in the exposure assessment step; see Table 1); guideline development; policy development; and health estimation in occupational health and safety. The tool could potentially also be applied to assess RoB in prevalence studies of exposure to other risk factors (e.g. environmental exposures or life style factors), but this should be carefully considered and, if necessary, the tool may need modification.

## Financial support

All authors are salaried staff members of their respective institutions. The publication was prepared with financial support from the 10.13039/100004423World Health Organization cooperative agreement with the Centres for Disease Control and Prevention National Institute for Occupational Safety and Health of the United States of America on implementing Resolution WHA 60.26 “Workers' Health: Global Plan of Action” (Grant 1 E11 OH0010676-02).

## Sponsors

The sponsors of this tool development are the World Health Organization and the International Labour Organization.

## Author contributions

Conceived the idea: FP, Paul Whaley (Associate Editor for Systematic Reviews, Environment International; Lancaster Environment Centre, Lancaster University, United Kingdom).

Developed first prototype of tool: FP.

Led all aspects of tool development: FP, DM.

Contributed to tool development: LAB, DG, LG, TL, AM, RLM, SLN, DP, PTJS, VS, EKS, KS, TT, YU, EvD, TJW.

Developed methodology and facilitated pilot testing: FP.

Pilot tested the tool: CB, AD, DG, LG, TL, AM, MBSP, PS, DT.

Analysed results of pilot tests: FP, DM, DS.

Wrote first draft and led development of manuscript: FP.

Contributed to manuscript writing and development: CB, LAB, AD, DG, LG, TL, AM, RLM, SLN, DP, MBSP, PTJS, VS, EKS, KS, PS, TT, DT, YU, EvD, TJW, DM.

The authors alone are responsible for the views expressed in this article and they do not necessarily represent the views, decisions or policies of the institutions with which they are affiliated.

## Declaration of Competing Interest

Susan L. Norris is a member of the GRADE working group.

## Appendices 1, 2 and 3 Supplementary data

Supplementary data Supplementary material Image 1

## Footnotes

Appendices 1, 2 and 3 Supplementary data to this article can be found online at https://doi.org/10.1016/j.envint.2019.105039.
