# Supplementary material for: RoB-SPEO: A tool for assessing risk of bias in studies estimating the prevalence of exposure to occupational risk factors from the WHO/ILO Joint Estimates of the Work-related Burden of Disease and Injury
Source: Environ Int. 2020 Feb;135:105039. doi: 10.1016/j.envint.2019.105039 (PMC7479507; doi:10.1016/j.envint.2019.105039)
Supplement: Supplementary data — Supplementary material [file mmc1.docx]

**Supplementary data for manuscript:**

**Pega et al.: RoB-SPEO: A tool for assessing risk of bias in studies estimating the prevalence of exposure to occupational risk factors from the WHO/ILO Joint Estimates of the Work-Related Burden of Disease and Injury**

[**Appendix 1: RoB-SPEO tool for assessing risk of bias in non-randomized studies estimating the prevalence of exposure to occupational risk factors** 2](#_Toc14075118)

[**Appendix 2: Selected excluded tools and the rationale for their exclusion** 19](#_Toc14075119)

[**Appendix 3: Domains from existing tools not included in the new tool and the reasons for this exclusion** 20](#_Toc14075120)

[**Reference list for all appendices** 21](#_Toc14075121)

# **Appendix 1: RoB-SPEO tool for assessing risk of bias in non-randomized studies estimating the prevalence of exposure to occupational risk factors**

**RoB-SPEO tool for assessing risk of bias in non-randomized studies estimating the prevalence of exposure to occupational risk factors**

**Instructions**

*Throughout the tool, instructions are always marked in Italics.*

*Please evaluate each individual study for all following eight risk of bias domains, each of which is specified by one key prompting question to guide your assessment.*

*For each domain, rate the risk of bias as “low”; “probably low”; “probably high”; or “high”. Provide one rating for each domain.*

*If the information you require to rate risk of bias for a domain is not reported, choose the category “no information”.*

*Report both your rating and a detailed justification for your rating.*

*If you can with confidence judge the direction of bias based on available evidence, report this judgment and the supporting evidence. If you cannot with confidence make this judgement, please do not guess the direction of the bias; rather, state that the direction of bias is unclear or cannot be determined.*

*Some risks of bias could potentially be captured in considerations for two or more domains. If this situation occurs, select the most appropriate domain to evaluate this risk of potential bias, to avoid counting the same risk of bias twice. For example, if you could consider the missingness of exposure data while assessing selection bias and bias due to incomplete exposure data, then consider missingness only in the domain you consider most appropriate and avoid double-counting risk.*

**Domain 1: Bias in selection of participants into the study**

**Prompting question**

Could the exposure status (or level) assessed (or assigned) in the study sample not represent exposure in the target population?

**Definitions**

| Bias in selection of participants into the study | Bias in selection of participants into the study (commonly called selection bias) is the bias due to systematic differences between the characteristics of the study sample and those of the target population ^1^. |
| --- | --- |
| Study sample | The sample of individuals participating in the study. Some individuals, who have been invited to participate in the study, may have opted to not participate, and if they were selected out of the study sample differentially by exposure level, this may have introduced selection bias (i.e. selection bias due to non-participation in the study). Some study participants may have opted to not participate in the exposure assessment, and if they are selected out of the assessment differentially by exposure level, this non-participation in the exposure assessment may have introduced selection bias (i.e. selection bias due to non-participation in the *exposure assessment*). If non-participants in the study or the exposure assessment have provided information on their reasons for non-participation, this may provide clues as to whether the non-participation may have introduced selection bias. |
| Target population | The population for which the authors of the study sought to assess exposure |
| Direct evidence | Empirical evidence specifically from the study you are assessing. For example, the study records of the study you are assessing provides an empirical analysis that demonstrates that the study had a risk of selection bias. |
| Indirect evidence | Empirical evidence from another study or other similar studies that you consider transferable to the study you are assessing. For example, the study records of the study you are assessing provide no empirical analysis that demonstrates that the study had a risk of selection bias, but empirical evidence was provided in similar studies that have demonstrated a risk of bias. |

*Before you start your considerations and assessment for this domain, please clearly define:*

- *The study population of the study that you are assessing.*
- *The target population for the study that you are assessing.*

*Record your definitions in the table for recording at the end of the sections for this domain.*

**Considerations**

The following list presents selected considerations that may systematically influence selection in a way that may introduce bias. These considerations should be interpreted as suggestions only and not as a comprehensive list or a checklist. Your assessment should always focus on the risk that the exposure in the study sample does not represent the exposure in the target population.

If any of the following might have led to differences in exposure levels between the study sample and the target population this may suggest risk of bias:

1. Representativeness of study sample
   1. The study’s eligibility criteria systematically excluded or included participants in the study sample (see Definitions) such that exposure in the study sample may not represent exposure in the target population (see Definitions).
   2. The sampling frame from which the study sample was drawn is not representative of the target population.
   3. The study sample was either not a full census of the target population, or not sampled randomly or quasi-randomly
   4. Study data demonstrate that the study sample and the target population are different in characteristics that may relate to exposure levels (e.g., occupations, workplace, employment conditions, sociodemographic characteristics and occupational health and safety measures, including presence of occupational health and safety management systems, compliance with occupational health and safety regulations and use of personal protective protection).
2. Missing participants or exposure measurements (*Only penalize the study for missing participants or exposure measurements once: either here in domain 1 on Bias in selection of participants into the study or in the domain 4 on Bias due to incomplete exposure data.)*
   1. A large proportion of persons invited to participate in the study did not participate (i.e. low rate of participation in the study).
   2. The proportion of persons invited to participate in the study who did participate was different across exposure levels or reasonable proxies for exposure levels (e.g. occupations).
   3. The reasons for non-participation in the study were either unclear, unacceptable or inappropriate (e.g. workers reported not participating, because they feared retaliation from their employers).
   4. A large proportion of study participants did not participate in the exposure assessment (i.e. low rate of participation in exposure assessment).
   5. The proportion of study participants who participated in the exposure assessment was different across exposure levels, for example due to variations in the workforce employed in different operations within an industry.

*If feasible, rather than relying solely on information from the principal record of the study, please also access and consider information reported in other records from or about the study.*

**Criteria for rating**

Criteria for a rating of LOW risk of bias (i.e., the answer to Question 1 above is: “No”):

You judge the risk to be low, because:

The study records provide sufficient detail for assessing risk of bias, including the: target population; study sample; criteria for eligibility for individuals to participate in the study; sampling, recruitment and enrolment procedures; rate of participation in the study; and rate of participation in the exposure assessment. The data reported in the study records are sufficiently detailed to support the conclusion that exposure in the study sample is representative of exposure in the target population, with similar characteristics reported for both the study sample and the target population.

OR

The descriptions and/or data as indicated above do suggest the potential for selection bias. However, adequate information was given that you can judge any potential selection as not differential across sub-groups defined by exposure levels.

OR

The descriptions and/or data as indicated above suggest the potential for selection, and there was no information suggesting that potential selection was not differential across sub-groups defined by exposure levels. However, drivers of selection were well-understood, these drivers of selection were measured in the data set, and appropriate post-hoc statistical methods were used to control for potential selection bias.

Criteria for the rating of PROBABLY LOW risk of bias (i.e., the answer to Question 1 above is: “Probably no”):

Information about selection does not suffice to permit you to judge the risk of bias to be low. Indirect evidence (see Definitions) suggests that inclusion/exclusion criteria, recruitment and enrolment procedures, and participation/response rates were similar across groups as described by the criteria for a rating of low risk of bias.

Criteria for the rating of PROBABLY HIGH risk of bias (i.e., the answer to Question 1 above is: “Probably yes”):

Information about selection does not suffice to permit you to judge the risk of bias to be high. Indirect evidence suggests that inclusion/exclusion criteria, recruitment and enrolment procedures, and participation/response rates differed across groups, as described by the criteria for a rating of high risk of bias.

Criteria for the rating of HIGH risk of bias (i.e., the answer to Question 1 above is: “Yes”):

The descriptions of the target population, inclusion/exclusion criteria, recruitment and enrolment procedures, participation/response rates and/or data on the distribution of relevant study sample and population characteristics suggest that the risk of selection bias was substantial;

AND

No information was provided to indicate that potential selection was not differential across sub-groups defined by exposure level;

AND

No appropriate post-hoc techniques were used to control for potential selection bias.

Criteria for the rating of NO INFORMATION (i.e., the answer to Question 1 above is: “There is insufficient information for assessing the risk of bias.”):

There is insufficient information for assessing the risk of bias.

**Table to record your assessment**

*Please complete all items of this table to record your assessment for this domain.*

| **Reporting item** | **Your assessment** |
| --- | --- |
| Definition of target population | *Add definition.* |
| Definition of the study population | *Add definition.* |
| Data extracted from study record(s) to support your assessment for this domain | *Extract text verbatim from the study record(s).* |
| Rating | *Select one of the following five standard categories:*   1. *Low* 2. *Probably low* 3. *Probably high* 4. *High* 5. *No information* |
| Justification for rating | *Justify your rating.* |

**Domain 2: Bias due to a lack of blinding of study personnel**

**Prompting question**

Could study personnel have known the exposure status (or level) or other characteristics of study participants and, if yes, could this knowledge have influenced how they conducted the exposure assessment (or assignment)?

**Definitions**

| Bias due to a lack of blinding of study personnel | Bias due to a lack of blinding of study personnel (commonly called performance bias) is the bias that arises due to a lack of blinding of exposure assessors and other study personnel to relevant participant characteristics (e.g. disease status) that leads to exposure assessment that differs depending on participant characteristics. For example, if an exposure assessor is not blinded to the disease status of a study participant, and if the exposure is a known risk factor for the disease, the lack of blinding of the exposure assessor may influence the assessor to measure exposure differentially for participants with the disease and those without the disease. |
| --- | --- |

**Criteria for rating**

Criteria for a rating of LOW risk of bias (i.e., the answer to Question 2 above is: “No”):

Exposure assessors and study personnel were blinded to relevant participant characteristics, and the blinding was probably not broken.

OR

Exposure assessors and study personnel were not blinded to relevant participant characteristics. However, this lack of blinding is unlikely to have influenced the exposure assessment (e.g., it was likely that exposure was systematically assessed similarly across sub-groups defined by participants characteristics);

Criteria for the rating of PROBABLY LOW risk of bias (i.e., the answer to Question 2 above is: “Probably no”):

The information on blinding is insufficient to permit a rating of low risk of bias. However, indirect evidence suggests that the exposure assessors and study personnel were adequately blinded or blinding was unlikely to influence exposure assessment, as described by the criteria for a rating of low risk of bias.

Criteria for the rating of PROBABLY HIGH risk of bias (i.e., the answer to Question 2 above is: “Probably yes”):

The information on blinding is insufficient to permit a rating of high risk of bias. However, indirect evidence suggests that the exposure assessors and study personnel were not adequately blinded or blinding was likely to influence exposure assessment, as described by the criteria for a rating of high risk of bias.

Criteria for the rating of HIGH risk of bias (i.e., the answer to Question 2 above is: “Yes”):

Exposure assessors and study personnel were not blinded or were incompletely blinded, and the exposure assessment was likely to be influenced by the lack of blinding (e.g., exposure was systematically assessed differentially for sub-groups defined by participant characteristics).

OR

Blinding of exposure assessors and study personnel was attempted, but may have been broken, and the breaking of the blinding may have introduced bias.

Criteria for the rating of NO INFORMATION (i.e., the answer to Question 1 above is: “There is insufficient information for assessing the risk of bias.”):

There is insufficient information for assessing the risk of bias.

**Table to record your assessment**

*Please complete all items of this table to record your assessment for this domain.*

| **Reporting item** | **Your assessment** |
| --- | --- |
| Data extracted from study record(s) to support your assessment for this domain | *Extract text verbatim from the study record(s).* |
| Rating | *Select one of the following five standard categories:*   1. *Low* 2. *Probably low* 3. *Probably high* 4. *High* 5. *No information* |
| Justification for rating | *Justify your rating.* |

**Domain 3: Bias due to exposure misclassification**

**Prompting question**

Could the methods used for assessing (or assigning) exposure have over- or under-estimated exposure?

**Definitions**

| Bias due to exposure misclassification | Bias due to exposure misclassification is “erroneous [and systematic] classification of an individual, a value, or an attribute into a [exposure] category other than that to which it should be assigned”, leading to under- or over estimation of prevalence of exposure status (or level) ^1^. For example, in the assessment of an exposure that seeks to assess whether a study participant was exposed below an exposure level indicating an occupational exposure (“unexposed”) or above this exposure level (“exposed”), if study participants who were exposed below this exposure level were systematically misclassified as “exposed”, this suggests the presence of misclassification bias. |
| --- | --- |

**Considerations**

The following list presents selected considerations that may systematically influence exposure classification in a way that may introduce misclassification bias. These considerations are suggestions only; they should not be used to formulate a score or as a checklist.

1. Exposure assessment and/or assignment (the assessment of an exposure based on its determinants)
   1. The exposure definition was not appropriate or an appropriate exposure definition was applied inconsistently.
   2. The exposure assessment and/or assignment was either based on exposure or job/occupation self-reported by the participant for whom the exposure was assessed or used a group-based approach (e.g. internal or external job exposure matrix), as opposed to being measured directly and objectively.
   3. In self-reported exposure assessment, study participants were aware of their own exposure status (e.g. from previous measurements taken).
   4. The exposure assessed was dependent on study participants’ characteristics (e.g. occupation, social factors and/or disease status).
   5. The exposure assessment was based on exposure measurements collected from the general background at the workplace (e.g. population level by far-field sampling), as opposed to by personal sampling (at an individual level).
   6. The exposure assessment captured any non-occupational (e.g. environmental) exposure to the occupational risk factor, when it should have exclusively captured occupational exposure to it.
   7. The time and duration of exposure measurement only covered parts of the working period, when they should have covered the entire working period.
   8. The exposure assessment did not accurately capture the use of personal protective equipment, as implemented among the study participant being assessed, or other exposure mitigating strategies, as implemented in the workplace of the study participant being assessed.
   9. If technical devices were used for exposure assessment, the technical requirements of the devices used to perform the exposure measurement did not meet specific and established standards (e.g. devices were not properly maintained and calibrated).
   10. The method of exposure assessment has either been shown to be ineffective, or it has not been validated, for the industrial sector and work practice of interest.
2. Data collection: Different exposure assessment and/or assignment modes were used for measuring exposure among the study participants (e.g. one group was assessed using face-to-face survey, whereas a second group was assessed using computer-assisted survey).
3. Sample size: The total study sample and/or the study sub-samples for each exposure level were judged to be too small to produce reliable estimates, ideally based on a formal power calculation total

**Criteria for rating**

Criteria for a rating of LOW risk of bias (i.e., the answer to Question 3 above is: “No”):

*Direct* evidence suggests that the method adopted in the study for the exposure assessment and assignment produces valid, accurate and reliable exposure data, ideally based on direct quantitative exposure measures.

OR

*Indirect* evidence suggests that the method used for assessing and assigning exposure produces valid, reliable and accurate measures of the exposure of interest. For example, a less established or less direct method was used, but there is indirect evidence that it performs equally well as a valid, reliable and accurate method or there is no validated exposure assessment or assignment method.

Criteria for the rating of PROBABLY LOW risk of bias (i.e., the answer to Question 3 above is: “Probably no”):

Insufficient information exists about the exposure assessment and assignment method to permit you to rate the risk of bias as low. However, there is *indirect* evidence that the methods were valid, accurate and reliable, as described by the criteria for a rating of low risk of bias:

*Indirect* evidence suggests that the exposure was assessed using validated methods that directly measure exposure.

OR

Exposure was assessed using semi-objective or subjectively reported measures (e.g., questionnaire or occupational exposure measurement by a certified occupational health and safety professional) that have been validated or empirically shown to be consistent with methods that directly measure exposure (i.e., inter-methods validation: one method vs. another).

Criteria for the rating of PROBABLY HIGH risk of bias (i.e., the answer to Question 4 above is: “Probably yes”):

You judge there to be insufficient information about the exposure assessment and assignment methods to permit a rating of high risk of bias, but there is *indirect* evidence that suggests that methods were not robust, as described by the criteria for a rating of high risk of bias:

*Indirect* evidence suggests that the exposure was assessed using not fully validated methods that directly measure exposure.

OR

There is evidence that the exposure was assessed using indirect measures that have not been validated or empirically shown to be consistent with methods that directly measure exposure (e.g., non-validated questionnaire, job-exposure matrix or self-report without validation).

OR

Insufficient information is provided about the exposure measurement method, including validity and reliability, but there is evidence for concern about the exposure measurement method used.

Criteria for the rating of HIGH risk of bias (i.e., the answer to Question 4 above is: “Yes”):

*Direct* evidence suggests that the exposure was assessed using methods with poor validity.

OR

Evidence suggests an exposure misclassification (e.g., differential recall of self-reported exposure).

OR

It is unclear how exposure assessment was performed and exposure measures were obtained, and there is evidence for concern about the exposure measurement method used.

Criteria for the rating of NO INFORMATION (i.e., the answer to Question 1 above is: “There is insufficient information for assessing the risk of bias.”):

There is insufficient information for assessing the risk of bias.

**Table to record your assessment**

*Please complete all items of this table to record your assessment for this domain.*

| **Reporting item** | **Your assessment** |
| --- | --- |
| Data extracted from study record(s) to support your assessment for this domain | *Extract text verbatim from the study record(s).* |
| Rating | *Select one of the following five standard categories:*   1. *Low* 2. *Probably low* 3. *Probably high* 4. *High* 5. *No information* |
| Justification for rating | *Justify your rating.* |

**Domain 4: Bias due to incomplete exposure data**

**Prompting question**

Could data on exposure status (or level) be incomplete for eligible participants?

**Definitions**

| Bias due to incomplete exposure data | Bias due to incomplete exposure data is the biases that arises from exposure data missing in a way that the exposure assessment is differential by exposure status (or level) in the target population (i.e. not random). If exposure data are incomplete (i.e., some exposure data are missing), and especially if these incomplete exposure data are missing in a way that the exposure assessment is differential by level of exposure in the target population (i.e. missing not at random), this presents a risk of bias from incomplete exposure data. For example, if 80% of study participants who were exposed to the occupational risk factor did not participate in the exposure assessment, but 100% of unexposed study participants participated in the exposure assessment, then this may have introduced bias from incomplete exposure data. |
| --- | --- |

**Considerations**

The risk of bias from incomplete exposure data depends on several factors, including the amount and distribution of missing data across levels of exposure, the reasons for missing exposure assessments, the amount of difference in exposure between participants with and without exposure data, the methods used by study authors to address missing data, and the context of the study with incomplete exposure data.

**Criteria for rating**

Criteria for a judgment of LOW risk of bias (i.e., the answer to Question 4 above is: “No”):

Non-participation in the study, non-participation in the exposure assessment and – for repeated measures studies – attrition of study participants over time were so low that you judge them to not meaningfully influence the exposure assessment.

AND

There were no or minimal incomplete exposure data or any incomplete exposure data have been imputed using appropriate methods.

Criteria for the judgment of PROBABLY LOW risk of bias (i.e., answer: “Probably no”):

There is insufficient information about the completeness of exposure data to permit a judgment of low risk of bias, but there is indirect evidence which suggests incomplete exposure data were adequately addressed, as described by the criteria for a judgment of low risk of bias.

OR

Indirect evidence suggests that exposure data are missing in the target population (e.g. there is evidence that exposure data are missing non-differentially by participant characteristics that may approximate the exposure, such as occupation, workplace, sociodemographic characteristics and/or health outcomes).

Criteria for the judgment of PROBABLY HIGH risk of bias (i.e., answer: “Probably yes”):

There is insufficient information about incomplete exposure data to permit a judgment of high risk of bias, but there is indirect evidence that incomplete exposure data were not adequately addressed, as described by the criteria for a judgment of high risk of bias.

OR

Indirect evidence suggests that exposure data are missing in the target population (e.g. there is evidence that exposure data are missing differentially by participant characteristics that may approximate the exposure).

Criteria for the judgment of HIGH risk of bias (i.e., answer: “Yes”):

Participation in the study was so low that this could have introduced bias.

OR

Participation in the exposure assessment among study participants was so low that this could have introduced bias.

OR

Direct evidence suggests that exposure data are missing in the target population.

OR

The incomplete exposure data have been imputed using inappropriate methods.

Criteria for the rating of NO INFORMATION (i.e., the answer to Question 1 above is: “There is insufficient information for assessing the risk of bias.”):

There is insufficient information for assessing the risk of bias.

**Table to record your assessment**

*Please complete all items of this table to record your assessment for this domain.*

| **Reporting item** | **Your assessment** |
| --- | --- |
| Data extracted from study record(s) to support your assessment for this domain | *Extract text verbatim from the study record(s).* |
| Rating | *Select one of the following five standard categories:*   1. *Low* 2. *Probably low* 3. *Probably high* 4. *High* 5. *No information* |
| Justification for rating | *Justify your rating.* |

**Domain 5: Bias due to selective reporting of exposures**

**Prompting question**

Could relevant exposures or exposure categories be selectively not reported?

**Definitions**

| Bias due to selective reporting of exposures | Bias due to selective exposure reporting is the systematic difference arising from selective reporting of exposures or exposure categories. |
| --- | --- |

*Rather than relying solely on information from the principal record of the study, please also use other records from or about the study. You should search for and use especially a pre-registered study protocol and/or website that contains registered details of the study, to enable you to compare the planned exposures and exposure categories with those for which results were reported.*

**Criteria for rating**

Criteria for a judgment of LOW risk of bias (i.e., the answer to Question 5 above is: “No”):

There was a pre-published protocol, and the prevalence of all exposures and exposure categories has been reported as pre-specified in the protocol.

Criteria for the judgment of PROBABLY LOW risk of bias (i.e., the answer to Question 5 above is: “Probably no”):

There is insufficient information about selective exposure reporting to permit a judgment of low risk of bias, but there is indirect evidence which suggests the study was free of selective exposure reporting, as described by the criteria for a judgment of low risk of bias.

Criteria for the judgment of PROBABLY HIGH risk of bias (i.e., the answer to Question 5 above is: “Probably yes”):

There is insufficient information about selective exposure reporting to permit a judgment of high risk of bias, but there is indirect evidence which suggests the study was not free of selective exposure reporting, as described by the criteria for a judgment of high risk of bias.

Criteria for the judgment of HIGH risk of bias (i.e., answer: “Yes”):

There was a pre-published protocol, but no results were reported for one or more primary exposures and/or exposure categories pre-specified for analysis in the protocol (unless clear justification for their non-reporting is provided).

OR

There was a pre-published protocol, but results were reported for one or more exposures or exposure categories that had not been pre-specified in the protocol (unless clear justification for their reporting is provided).

OR

There was a pre-published protocol, but results were reported incompletely for one or more pre-specified exposures or exposure categories.

OR

There was no pre-published protocol, and there was direct evidence that the study was not free of selective exposure reporting.

Criteria for the rating of NO INFORMATION (i.e., the answer to Question 1 above is: “There is insufficient information for assessing the risk of bias.”):

There is insufficient information for assessing the risk of bias.

**Table to record your assessment**

*Please complete all items of this table to record your assessment for this domain.*

| **Reporting item** | **Your assessment** |
| --- | --- |
| Data extracted from study record(s) to support your assessment for this domain | *Extract text verbatim from the study record(s).* |
| Rating | *Select one of the following five standard categories:*   1. *Low* 2. *Probably low* 3. *Probably high* 4. *High* 5. *No information* |
| Justification for rating | *Justify your rating.* |

**Domain 6: Bias due to conflict of interests**

**Prompting question**

Could the study and/or one or more study authors have received support from entities with potential interests in the exposure assessed (or assigned)?

**Definitions**

| Bias due to conflicts of interest | Bias due to conflicts of interest is the bias introduced if financial and other interests influence the design, conduct, data collection, analysis and/or reporting of a study ^2^. |
| --- | --- |

The standard International Committee of Medical Journal Editors (ICMJE) Form for Disclosure of Potential Conflicts of Interest ^2^ categorises four types of potential (i.e. real or perceived) conflicts of interest:

1. “The Work Under Consideration for Publication”: “Receipt of payment or services from a third party (government, commercial, private foundation, etc.) for any aspect of the submitted work (including but not limited to grants, data monitoring board, study design, manuscript preparation, statistical analysis, etc.)”. (p2) Conflicts of interest of this type could include: industry grants (issued or pending); payment for manuscript preparation; royalties; payment for development of educational presentations; and travel reimbursement.
2. “Relevant financial activities outside the submitted work”: “Financial relationships (regardless of amount of compensation) with […] entities as described in the instructions [that is, government agency, foundation, commercial sponsor, academic institution, etc. ] […] present during the 36 months prior to publication”. (p2) This could include: concurrent or former board membership; concurrent or former consultancy work; concurrent or former industry employment; expert testimony; industry grants (issued or pending); payment for lectures including service on speakers bureaus; royalties; payment for development of educational presentations; stock or stock options; and travel reimbursement, or other relations with relevant industries.
3. “Intellectual Property -- Patents & Copyrights”: “patents, whether planned, pending or issued, broadly relevant to the work”. (p2)
4. “Other relationships not covered above”: “other relationships or activities that readers could perceive to have influenced, or that give the appearance of potentially influencing, what […] [the author or authors] wrote in the submitted work.” (p2). Conflicts of interest of this type could include: intellectual interests; membership in interest groups; and employment for a not-for-profit organization with interests (e.g. employers’ associations or trade unions).

**Criteria for rating**

Criteria for a rating of LOW risk of bias (i.e., the answer to Question 6 above is: “No”):

There is no evidence that the study conduct or reporting was influenced by a competing interest.

Criteria for the rating of PROBABLY LOW risk of bias (i.e., the answer to Question 6 above is: “Probably no”):

There is insufficient information to permit a rating of low risk of bias, but there is *indirect* evidence that the study conduct or reporting was not influenced by a competing interest.

Criteria for the rating of PROBABLY HIGH risk of bias (i.e., the answer to Question 6 above is: “Probably yes”):

There is insufficient information to permit a rating of high risk of bias, but there is *indirect* evidence that the study conduct or reporting was influenced by a competing interest.

Criteria for the rating of HIGH risk of bias (i.e., the answer to Question 6 above is: “Yes”):

There is evidence that the study conduct or reporting was influenced by a competing interest.

Criteria for the rating of NO INFORMATION (i.e., the answer to Question 1 above is: “There is insufficient information for assessing the risk of bias.”):

There is insufficient information for assessing the risk of bias.

**Table to record your assessment**

*Please complete all items of this table to record your assessment for this domain.*

| **Reporting item** | **Your assessment** |
| --- | --- |
| Data extracted from study record(s) to support your assessment for this domain | *Extract text verbatim from the study record(s).* |
| Rating | *Select one of the following five standard categories:*   1. *Low* 2. *Probably low* 3. *Probably high* 4. *High* 5. *No information* |
| Justification for rating | *Justify your rating.* |

**Domain 7: Bias due to differences in numerator and denominator**

**Prompting question**

Could the definition and/or counting of persons contributing to the numerator differ from those contributing to the denominator in the ratio used to estimate prevalence?

**Definitions**

| Bias due to differences in numerator and denominator | Bias due to differences in numerator and denominator is the bias that arises when there is a mismatch of definition and/or counting of persons contributing to the numerator and the denominator in the ratio used to estimate prevalence ^3^. |
| --- | --- |

**Considerations**

Considerations for assessing the risk of bias due to differences in numerator and denominator include:

1. The definitions of the numerator and denominator were not comparable.
2. The numerator and denominator populations were counted differently.
3. The shortest prevalence period for the assessed exposure was too short (e.g. not at least one full working day).

**Criteria for rating**

Criteria for a rating of LOW risk of bias (i.e. the answer to Question 7 above is: “No”):

The numerator and denominator were clearly defined and counted correctly and/or the shortest prevalence period was appropriate (e.g. at least one full working day).

Criteria for the rating of PROBABLY LOW risk of bias (i.e., the answer to Question 7 above is: “Probably no”):

There is insufficient information to permit a rating of low risk of bias, but there is indirect evidence, which suggests the study was free of bias due to differences in numerator and denominator, as described by the criteria for a rating of low risk of bias.

Criteria for the rating of PROBABLY HIGH risk of bias (i.e., the answer to Question 7 above is: “Probably yes”):

There is insufficient information to permit a rating of high risk of bias, but there is indirect evidence, which suggests the study was not free of bias due to differences in numerator and denominator, as described by the criteria for a rating of high risk of bias.

Criteria for the rating of HIGH risk of bias (i.e., the answer to Question 7 above is: “Yes”):

The numerator and denominator are defined and/or counted differently, and/or the shortest prevalence period was inappropriate (e.g. too short to detect exposure).

Criteria for the rating of NO INFORMATION (i.e., the answer to Question 1 above is: “There is insufficient information for assessing the risk of bias.”):

There is insufficient information for assessing the risk of bias.

**Table to record your assessment**

*Please complete all items of this table to record your assessment for this domain.*

| **Reporting item** | **Your assessment** |
| --- | --- |
| Data extracted from study record(s) to support your assessment for this domain | *Extract text verbatim from the study record(s).* |
| Rating | *Select one of the following five standard categories:*   1. *Low* 2. *Probably low* 3. *Probably high* 4. *High* 5. *No information* |
| Justification for rating | *Justify your rating.* |

**Domain 8: Other bias**

**Prompting question**

Could the study have other problems that could have introduced bias?

**Definitions**

| Other biases | Other bias is any other bias specific to a particular study rather than applicable to all studies. |
| --- | --- |

**Criteria for rating**

Criteria for a rating of LOW risk of bias (i.e. the answer to Question 7 above is: “No”):

You judge the study to be free of other potential sources of bias.

Criteria for the rating of PROBABLY LOW risk of bias (i.e., the answer to Question 7 above is: “Probably no”):

There is insufficient information to permit a rating of low risk of bias, but there is indirect evidence, which suggests the study was free of other potential sources of bias.

Criteria for the rating of PROBABLY HIGH risk of bias (i.e., the answer to Question 7 above is: “Probably yes”):

There is insufficient information to permit a rating of high risk of bias, but there is indirect evidence, which suggests the study was not free of other potential sources of bias, as described by the criteria for a rating of high risk of bias.

Criteria for the rating of HIGH risk of bias (i.e., the answer to Question 7 above is: “Yes”):

The study carried a potential risk of bias related to its specific study design (e.g. sampling strategy was not adequate to cover the most relevant time of the year/season, industrial production level and/or route of exposure and/or there were technical difficulties resulting in potential loss of exposure sample integrity).

OR

The study conduct was affected by interim results (e.g. additional participants have been recruited from a sub-group that interim results show was more likely or less likely to be exposed to the occupational risk factor).

Criteria for the rating of NO INFORMATION (i.e., the answer to Question 1 above is: “There is insufficient information for assessing the risk of bias.”):

There is insufficient information for assessing the risk of bias.

**Table to record your assessment**

*Please complete all items of this table to record your assessment for this domain.*

| **Reporting item** | **Your assessment** |
| --- | --- |
| Data extracted from study record(s) to support your assessment for this domain | *Extract text verbatim from the study record(s).* |
| Rating | *Select one of the following five standard categories:*   1. *Low* 2. *Probably low* 3. *Probably high* 4. *High* 5. *No information* |
| Justification for rating | *Justify your rating.* |

# **Appendix 2: Selected excluded tools and the rationale for their exclusion**

| **Excluded tool** | **Rationale for exclusion** |
| --- | --- |
| Tool for assessing risk of bias in randomised trials ^4^ | This tool is for assessing randomized studies of interventions only, but our new tool seeks to assess non-randomized prevalence studies of exposure to occupational risk factors. |
| Revised tool for assessing risk of bias in randomized trials (RoB 2.0) ^5^ | This tool is for assessing randomized studies of interventions only, but our new tool seeks to assess non-randomized prevalence studies of exposure to occupational risk factors. |
| Tool for assessing risk of bias in non-randomized studies of interventions (ACROBATNRSI 1.0.0) ^6^ | This tool is for assessing randomized studies of interventions only, but our new tool seeks to assess non-randomized prevalence studies of exposure to occupational risk factors.  This tool is the predecessor of ROBINS-I ^7^, which is included in our review. |
| Newcastle-Ottawa scale for assessing the quality of non-randomized studies in meta-analyses ^8^ | This tool is for assessing risk of bias and quality of evidence of cohort studies and case-control studies only, but our new tool seeks to assess all prevalence studies of exposure to occupational risk factors. |
| Guidelines for Accurate and Transparemnt Heath Estimates Reporting (GATHER) ^9,10^ | Although GATHER outlines selected RoB categories for reporting input data, it is a reporting guideline, not a tool for assessing risk of bias. |

# **Appendix 3: Domains from existing tools not included in the new tool and the reasons for this exclusion**

| **Existing tool** | **Domain not included in new tool** | **Reason for domain not being included in new tool** |
| --- | --- | --- |
| ROBINS-I tool ^7^ | Bias due to confounding | Exclusively relevant for studies estimating the effect of an intervention |
|  | Bias in classification of interventions |  |
|  | Bias due to deviations from intended interventions | Exclusively relevant for comparison to an ideal target intervention or exposure |
|  | Bias in measurement of outcomes | Exclusively relevant for assessment of a health outcome |
| Risk of bias instrument for non-randomized studies of exposures ^11^ | Bias due to confounding | Exclusively relevant for studies estimating the effect of an occupational or other exposure |
|  | Bias due to deviations from intended exposures | Exclusively relevant for comparison to an ideal target intervention or exposure |
|  | Bias in measurement of outcomes | Exclusively relevant for assessment of a health outcome |
| Navigation Guide tool (human evidence stream) ^12^ | Outcome misclassification bias | Exclusively relevant for assessment of a health outcome |
|  | Confounding | Exclusively relevant for studies estimating the effect of an occupational or other exposure |
| OHAT tool (cohort, case-control, cross-sectional and case series studies in human evidence stream) ^13^ | Confounding bias | Exclusively relevant for studies estimating the effect of an occupational or other exposure |

# **Reference list for all appendices**

1. Porta M. A Dictionary of Epidemiology. 6 ed. New York, NY: Oxford University Press; 2014.

2. International Committee of Medical Journal Editors. Conflict of Interest Disclosure Form. n.d. (accessed 12 December 2018).

3. Williams GM, Najman JM, Clavarino A. Correcting for numerator/denominator bias when assessing changing inequalities in occupational class mortality, Australia 1981 -2002. *Bulletin of the World Health Organization* 2006; **84**(3): 198-203.

4. Higgins JP, Altman DG, Gotzsche PC, et al. The Cochrane Collaboration's tool for assessing risk of bias in randomised trials. *Bmj* 2011; **343**: d5928.

5. Higgins J, Jelena Savović J, Page M, Sterne J. Revised Cochrane risk-of-bias tool for randomized trials (RoB 2). 2018. <https://sites.google.com/site/riskofbiastool/welcome/rob-2-0-tool/current-version-of-rob-2> (accessed 14 December 2018).

6. Sterne J, Higgins J, Reeves B. A Cochrane Risk Of Bias Assessment Tool for Non-Randomized Studies of Interventions (ACROBATNRSI), Version 1.0.0, 24 September 2014. Available from <http://www.riskofbias.info>. 2014.

7. Sterne JA, Hernan MA, Reeves BC, et al. ROBINS-I: a tool for assessing risk of bias in non-randomised studies of interventions. *Bmj* 2016; **355**: i4919.

8. Wells G, Shea B, O'Connell D, et al. The Newcastle-Ottawa Scale (NOS) for assessing the quality of nonrandomised studies in meta-analyses. 2019. <http://www.ohri.ca/programs/clinical_epidemiology/oxford.asp> (accessed 18 April 2019).

9. Stevens GA, Alkema L, Black RE, et al. Guidelines for Accurate and Transparent Health Estimates Reporting: the GATHER statement. *Lancet* 2016; **388**(10062): e19-e23.

10. The GATHER Working Group. The GATHER Statement: Explanation and Elaboration. Geneva: World Health Organization, 2016.

11. Morgan RL, Thayer KA, Santesso N, et al. A risk of bias instrument for non-randomized studies of exposures: A users' guide to its application in the context of GRADE. *Environ Int* 2019; **122**: 168-84.

12. Lam J, Sutton P, Padula AM, et al. Applying the Navigation Guide Systematic Review Methodology Case Study #6: Association between Formaldehyde Exposure and Asthma: A Systematic Review of the Evidence: Protocol. San Francisco, CA: University of California at San Francisco, 2016.

13. Office of Health Assessment and Translation. Handbook for Conducting a Literature-Based Health Assessment Using OHAT Approach for Systematic Review and Evidence Integration. Durham, NC: National Institute of Environmental Health Sciences; 2015.
